# Tree species-specific strategies of soil aggregation driven by SOC–GRSP coupling under nitrogen addition and precipitation reduction

**DOI:** 10.1371/journal.pone.0341117

**Published:** 2026-01-21

**Authors:** Mingxin Zhou, Yibo Li, Wei Liu, Chao Jia, Jiantao Hao

**Affiliations:** 1 Heilongjiang Institute of Construction Technology, Harbin, China; 2 Institute of Geographic Sciences and Natural Resources Research, Chinese Academy of Sciences, Beijing, China; 3 Heilongjiang Vocational Institute Ecological Engineering, Harbin, China; 4 Heilongjiang Polytechnic, Harbin, China; Zhejiang Agriculture and Forestry University: Zhejiang A and F University, CHINA

## Abstract

Soil structural stability underpins ecosystem function, yet how nitrogen (N) enrichment and precipitation reduction jointly regulate glomalin-related soil proteins (GRSP) and aggregate formation in temperate forests remains poorly understood. This knowledge gap limits predictions of soil carbon persistence under global change. A factorial field experiment was conducted in an old-growth temperate forest with four treatments (CK, + N, –P, + N–P) across three dominant tree species. Rhizosphere soils were analyzed for total and easily extractable GRSP (T-GRSP, EE-GRSP), aggregate-size distribution, and physicochemical properties. Random forest modeling and structural equation modeling (SEM) were used to identify key regulatory pathways. N addition significantly increased EE-GRSP (3.92–5.74 mg g ⁻ ¹) and macroaggregates (4–8 mm: 21.6%–34.8%), while precipitation reduction reduced EE-GRSP (by 36.5%) and increased microaggregates (0.053–0.25 mm: + 29.3%). soil organic carbon (SOC) was strongly and positively correlated with EE-GRSP (R² = 0.69–0.63), T-GRSP (R² = 0.82–0.77), MWD (R² = 0.85–0.67), and GMD (R² = 0.84–0.72). Random forest identified EE-GRSP and SOC as dominant predictors of aggregate stability. SEM revealed that SOC regulated GRSP and MWD through NH₄ ⁺ –N and SWC (Fig. 2–5). Our findings highlight a coupled “carbon–protein–structure” pathway in regulating soil aggregation. The regulatory effects of N and water are both species-specific and pathway-integrated, emphasizing the role of SOC-mediated GRSP dynamics in sustaining soil physical integrity under climate perturbations.

## 1. Introduction

Soil structural stability is a fundamental component of ecosystem resilience, regulating water retention, root anchorage, nutrient cycling, and long-term carbon sequestration [1,2]. It emerges from complex interactions among physical aggregation processes, chemical bonds, and biologically mediated mechanisms [3,4]. Among the biological drivers, microbial metabolites and root-associated carbon inputs are particularly influential in binding soil particles and promoting macroaggregate formation [5,6]. However, this finely balanced system is increasingly disrupted by global environmental changes, particularly the twin pressures of elevated N deposition and reduced precipitation. N enrichment, driven by anthropogenic emissions, alters soil nutrient stoichiometry, root exudation patterns, and microbial metabolic activity, while precipitation reduction intensifies water stress, suppresses microbial functions, and constrains organic matter stabilization [7,8]. These perturbations impose non-linear and potentially antagonistic effects on soil carbon–microbe–structure linkages [9,10]. Despite growing attention, the integrated impacts of N and water availability on soil aggregation pathways—particularly those mediated by active microbial byproducts—remain poorly understood in forest ecosystems, limiting predictive capacity in the context of climate change.

Current understanding of soil aggregation dynamics under global change remains constrained by four major research gaps. First, most studies have focused on bulk surface soils (e.g., 0–5 cm), neglecting depth-specific variation in microbial activity, carbon inputs, and aggregation processes, thereby limiting our understanding of vertical heterogeneity in soil structural regulation [11,12]. Second, N effects are often assessed using a single chemical form, overlooking the distinct biogeochemical behaviors of ammonium and nitrate, which constrains insight into form-dependent microbial responses [13]. Third, many investigations treat microbial and plant components in isolation, failing to quantify how plant–microbe interactions co-regulate glomalin production and aggregate formation, especially under concurrent nutrient and water perturbations [14,15]. Fourth, limited attention has been given to interspecific variation in root traits and carbon exudation strategies, despite their known influence on rhizosphere microbial activity and soil structural feedbacks [16,17]. These knowledge gaps hinder our ability to identify trait-based mechanisms that govern soil aggregation and resilience under multifactorial environmental change.

To address these limitations, we established a field experiment in a temperate forest using a factorial design that manipulates N addition and precipitation reduction. Three co-occurring woody species—*Pinus koraiensis*, *Tilia amurensis*, and *Fraxinus mandshurica*—were selected to represent contrasting ecological strategies and root-associated carbon allocation patterns. Soil samples were analyzed for GRSP, both T-GRSP and EE-GRSP fractions, along with SOC, aggregate size distribution, and water content. By integrating species-level analysis with SEM, the study enables mechanistic exploration of how GRSP fractions mediate macroaggregate stability in response to simultaneous N and water perturbations. This design bridges plant–microbe–soil interactions with ecosystem-scale structural outcomes and offers a process-based framework for evaluating soil resilience under global environmental stress.

This study investigates the impact of N addition and reduced precipitation on GRSP accumulation and soil aggregate stability in three dominant tree species (*P. koraiensis, T. amurensis,* and *F. mandshurica*). To address how N deposition and altered precipitation interactively shape soil structural resilience in temperate forests, this study investigates the species-specific and mechanistic responses of glomalin-related soil proteins (GRSP) and soil aggregation. **(H1)** We hypothesize that tree species differ in their regulation of GRSP production and soil aggregation in response to N enrichment and water limitation. Specifically, *P. koraiensis* is expected to promote GRSP-mediated macroaggregate formation under N addition, whereas *T. amurensis* and *F. mandshurica* are more susceptible to drought-induced GRSP reductions and microaggregate dominance. These interspecific differences are anticipated to reshape aggregate-size distribution and reflect divergent soil stabilization strategies under combined environmental stressors. **(H2)** We further hypothesize that environmental resource availability—particularly soil organic carbon (SOC) and water content (SWC)—synergistically regulates the microbial synthesis of easily extractable glomalin-related soil protein (EE-GRSP), which in turn mediates the formation and stabilization of soil macroaggregates. Specifically, SOC is expected to enhance fungal exudation by providing metabolic substrates, while SWC modulates exudation efficiency and protein persistence. Compared to total GRSP, EE-GRSP is predicted to exert a stronger and more immediate influence on aggregate binding due to its bioavailability and physicochemical reactivity. This pathway—linking carbon–water dynamics to EE-GRSP accumulation and structural stabilization—constitutes a mechanistic bridge between abiotic conditions and microbially mediated soil aggregation [18,19]. By elucidating this environmentally modulated pathway, we aim to uncover the biogeochemical feedbacks driving soil structural dynamics under concurrent nutrient enrichment and hydrological stress.

The findings have direct implications for forest soil management and conservation strategies, particularly in regions experiencing increased N deposition and changing precipitation patterns. Understanding the resilience of different tree species to these environmental drivers will inform sustainable soil management practices to enhance carbon sequestration and soil structural integrity in forest ecosystems.

## 2. Materials and methods

### 2.1. Study sites

The research was carried out in an ancient broad-leaved Korean pine mixed forest, which has a history of over 300 years. This forest is situated within the Changbai Mountains Natural Reserve in northeastern China, precisely at the coordinates 42°24′ N and 128°06′ E, and it stands at an altitude of 738 meters above sea level. The local climate belongs to the typical temperate-continental type. Winters here are cold and prolonged, while summers are warm but relatively short. On average, the region receives about 740 mm of precipitation annually, and more than 80% of this rainfall occurs between May and October. The annual mean temperature hovers around 3.6 °C, and during the growing season from May to October, the average temperature reaches 15 °C. The forest is dominated by tree species such as *Pinus koraiensis*, *Tilia amurensis* and *Fraxinus mandshurica*, with a tree density of 432 trees per hectar, although the stand is mixed rather than pure, each dominant species forms spatially clustered patches that enabled species-specific sampling without interference from adjacent heterospecific individuals.

### 2.2. Plot design

In May 2009, six 50 m × 50 m plots were randomly established within a forest ecosystem, each separated by ≥25 m buffer zones to minimize cross-interference. Historical data from the Chinese Ecosystem Research Network revealed drought-year precipitation averaged 550 mm – 30% below the 30-year mean of 740 mm. To study precipitation reduction impacts on C/N cycling, three plots were equipped with elevated (1 m) V-shaped high-transmittance panels intercepting 30% of through-fall (-P treatment), approximating a 200 mm·yr ⁻ ¹ reduction. These structures preserved air circulation while being removed during winter to avoid snow redistribution artifacts. The remaining three plots served as precipitation controls.

Simultaneously, each plot was bisected into paired 25 m × 50 m subplots for N manipulation. To prevent nutrient transfer between adjacent subplots, stainless-steel sheets (50 cm depth) were inserted along subplot borders. One subplot received monthly NH₄NO₃ applications (totaling 50 kg N·ha ⁻ ¹·yr ⁻ ¹, double regional deposition rates) via backpack sprayer during May-October growing seasons, dissolved in 40 L water per application. Control subplots received equivalent water volumes without N. This split-plot design generated four replicated treatments (n = 3): control (CK), N addition (+N), precipitation reduction (-P), and interactive effects of N addition and precipitation. (+N-P).

The experimental framework enabled isolation of precipitation and N drivers while addressing their potential interactions through hierarchical spatial partitioning-first splitting plots for precipitation manipulation, then subdividing for N treatments. Such nested designs are particularly valuable in ecological studies requiring multiple treatment combinations. The standardized 40 L carrier volume controlled for watering effects across treatments, while winter panel removal demonstrated adaptive methodology for seasonal climatic variables.

### 2.3. Sample collection

Rhizosphere soils associated with *P. koraiensis*, *F. mandshurica*, and *T. amurensis.* were collected following standardized protocols, and the nearest heterospecific tree was generally located more than 3–5 m from each sampling point, ensuring that measured traits reflected the focal species. For each tree species within every plot, three individual trees were randomly selected, with three replicate soil samples obtained per tree using a systematic approach. All samples were collected within 1 m of mature focal trees in monospecific patches, and locations containing visible heterospecific roots were excluded to avoid cross-species interference. Prior to sampling, the litter layer was carefully removed to minimize surface contamination. A stainless-steel auger (5 cm diameter, 10 cm depth) was used to extract subsurface soil from the 0–10 cm horizon, with subsequent removal of visible roots and rock fragments. Composite soil samples were homogenized and immediately stored under refrigeration at 4°C to preserve microbial activity until laboratory processing. Sub-samples were allocated for distinct analytical purposes: one aliquot was designated for GRSP, as GRSP is a key glycoprotein exuded by arbuscular mycorrhizal fungi that strongly contributes to soil aggregation and carbon stabilization [14,18], while another underwent physicochemical characterization (Table 1). Soil pH determination involved suspending 4 g of air-dried soil in deionized water (1:2.5 w/v ratio), followed by 30-minute equilibration and measurement using a calibrated pH meter (Sartorius PB-10, Germany) [20]. For inorganic N analysis, 10 g of fresh soil underwent 0.05 M K₂SO₄ extraction, with NH₄⁺ and NO₃ ⁻ concentrations subsequently determined via flow injection analysis (Bran-Luebbe AA3, Germany) [21]. Total organic carbon content was analyzed through high-temperature combustion using an elemental analyzer (Multi N/C 3100, Analytik Jena, Germany), operating at 680 °C for approximately 5 minutes to ensure complete oxidation of organic carbon [22]. Gravimetric soil moisture content was calculated from mass loss after oven-drying 10 g fresh soil at 105°C to constant weight [23].

**Table 1:** Symbols.

Traits	Abbreviation	Units	Description
Soil properties			
Soil organic carbon	SOC	g/kg	Organic – carbon content in soil
Soil water content	SWC	%	Moisture in fresh soil
pH	pH		Soil’s acidity – alkalinity level
Soil NH_4_^+^	NH_4_^+^ -N	mg/kg	Ammonium – nitrogen content in soil
Soil NO_3_^−^	NO_3_^−^-N	mg/kg	Nitrate – nitrogen content in soil
Soil aggregate traits			
Geometric mean diameter	GMD	mm	Geometric average diameter of soil aggregates
Mean weight diameter	MWD	mm	Average weight – based diameter of soil aggregates
GRSP			
Total glomalin related soil protein	T-GRSP	g/kg	Total amount of glomalin – related soil protein
Easily extractable glomalin related soil protein	EE-GRSP	g/kg	Easily – extractable glomalin – related soil protein

### 2.4. Determinations of GRSP

The extraction of T-GRSP and EE-GRSP from each composite soil sample followed the method presented by Gu (2024) [24]. For T-GRSP extraction, 0.25 g of soil sieved to 2 mm was placed in a centrifuge tube. Then, 2 ml of 50 mmol L ⁻ ¹ sodium citrate solution (with a pH of 8.0) was added. The tube was autoclaved at 121°C for 60 minutes. After autoclaving, it was centrifuged at 5000 revolutions per minute (rpm) for 10 minutes. In the case of EE-GRSP extraction, the same quantity (0.25 g) of the 2-mm-sieved soil was used. 2 ml of 20 mmol L ⁻ ¹ sodium citrate solution (pH 7.0) was added to the soil in the centrifuge tube. The tube was autoclaved at 121°C for 30 minutes and then centrifuged at 5000 rpm for 10 minutes to eliminate the remaining soil particles. After each extraction process, the supernatant was carefully poured off and stored at 4°C. Sodium citrate was added to the remaining soil for subsequent extractions until the supernatant became a pale-yellow color. To measure the GRSP, a UV spectrophotometer (Shimadzu UV-1780, JP) was used. Bovine serum albumin (BSA) was employed as the standard, and the absorbance values of GRSP were measured at a wavelength of 595 nm. We acknowledge that the Bradford-based assay quantifies an operationally defined GRSP fraction that may contain non-glomalin proteins and humic-derived compounds [25].

### 2.5. Separation of soil aggregates

Soil aggregates were fractionated using a wet-sieving procedure adapted from previous studies to maintain their natural structure under water-saturated conditions [26]. Briefly, field-collected soil was carefully broken apart by hand and passed through an 8-mm mesh to remove coarse roots and other large organic debris. We air-dried the sieved soil. A 50 g subsample was then positioned on a pre-wetted stack of sieves (0.053, 0.25, 1.0, 2.0 and 4.0 mm) that had been immersed in water overnight to ensure thorough wetting. The next day, the sieve stack was vertically oscillated with a 5-cm amplitude at a frequency of 30 cycles per minute for 10 minutes. This process yielded five aggregate-size fractions: 0.053–0.25 mm, 0.25–1 mm, 1–2 mm, 2–4 mm, and 4–8 mm. Each fraction was carefully collected, dried, and weighed to determine its proportional mass relative to the total. To assess aggregate stability, MWD and GMD were calculated by integrating the mass distribution and the midpoint diameter of each aggregate-size fraction [27].


$$MWD=\sum_{i=1}^{n}x_{i}\omega_{i}$$ (1)

$$GMD=exp[(\sum_{i=1}^{\text{n}}\omega_{i}\;lg\;x_{i})/(\sum_{i=1}^{n}\omega_{i})]$$ (2)

${\text{x}}_{i}$ is the average particle size of soil aggregates (mm), and $\omega_{\text{i}}$ is the relative contribution of various soil aggregate fractions to the total aggregate mass.

## 3. Statistical analysis

In the statistical analysis part, we adopted different methods to delve into various aspects of the data. Firstly, to assess data normality, the Kolmogorov-Smirnov test was employed, and Levene’s test was used to evaluate the homogeneity of variances. Because multiple trees were sampled within each plot, individual trees were treated as random subsamples rather than independent analytical replicates. Therefore, plot-level means were used as the analytical unit in subsequent analyses to ensure statistical independence among treatments. For detecting significant differences in soil aggregate size fractions, NH₄ ⁺ -N, NO₃ ⁻ -N, SWC, SOC, pH, MWD, GMD, T-GRSP, and EE-GRSP among different treatments, a one-way analysis of variance (ANOVA) was carried out. To understand the interactive effects of treatments and species, a two-way ANOVA was performed on MWD, GMD, T-GRSP, EE-GRSP, and soil aggregate size fractions. After obtaining the ANOVA results, Tukey’s Honestly Significant Difference (HSD) test was used for post-hoc comparisons, with a significance level set at p < 0.05. In the presentation of results, different lowercase letters were used to represent significant differences among treatments within the same aggregate size fraction, while different uppercase letters indicated significant differences among aggregate size fractions within the same treatment. Regression analysis was utilized to explore the relationships between SOC and both GRSP and soil aggregate traits. To investigate the associations between soil aggregate size fractions and soil physicochemical properties such as SOC, MWD, GMD, T-GRSP, and EE-GRSP, Pearson’s correlation analysis was conducted. A random forest (RF) model was implemented using the “randomForest” package in R to identify the key determinants of GRSP and soil aggregate stability indices. Each RF model consisted of 1,000 regression trees, and the importance of each predictor variable was assessed based on the mean decrease in accuracy. To ensure model robustness and avoid overfitting, ten-fold cross-validation was applied, and variable importance values were bootstrapped 500 times to estimate the mean importance and associated confidence intervals. The significance of each predictor was determined using permutation tests (p < 0.05, p < 0.01). To further clarify the causal relationships among soil physicochemical properties, GRSP, and aggregate stability, a structural equation modeling (SEM) approach was applied using the “lavaan” package in R. The SEM was constructed according to theoretical expectations and tested with maximum likelihood estimation (MLE). Model adequacy was evaluated using multiple fit indices, including the chi-square to degrees of freedom ratio (χ²/df), Comparative Fit Index (CFI), Root Mean Square Error of Approximation (RMSEA), and Standardized Root Mean Square Residual (SRMR). The final model exhibited an excellent fit with the observed data (χ²/df = 1.82, CFI = 0.963, RMSEA = 0.045, SRMR = 0.031), meeting the criteria of CFI > 0.90 and RMSEA/SRMR < 0.08. These results confirmed that the hypothesized causal relationships among SOC, GRSP, and soil environmental factors were statistically reliable and ecologically meaningful. All the statistical analyses were carried out in R (version 4.2.1, R Core Team, 2018).

## 4. Results

### 4.1. Soil properties

Soil physicochemical properties exhibited significant variations among treatments (Table 2). Soil pH was significantly lower under +N than under CK and –P, whereas SWC declined markedly in the –P and +N–P treatments compared with CK and +N across all species (Table 2). NH₄ ⁺ -N content was significantly higher in the + N treatment compared to other treatments across all species. NO₃ ⁻ -N content was significantly higher in the + N treatment and significantly lower in the -P treatment for all species. SOC content was significantly lower in the -P treatment than in CK and +N treatments in all species. In *T. amurensis* and *F. mandshurica*, SOC was significantly lower in the -P treatment compared to the + N-P treatment (Table 2).

**Table 2:** Soil properties at different treatments (means ± se).

Species	Type	CK	+N	-P	+N-P
*Pinus koraiensis*	pH	5.05 ± 0.57a	4.95 ± 0.11b	5.32 ± 0.3a	4.85 ± 0.45b
	SWC(%)	53.25 ± 6.22a	54.43 ± 5.56a	46.4 ± 3.37b	40.46 ± 5.08c
	NH_4_^+^-N (mg/kg)	5.37 ± 0.13b	8.02 ± 0.53a	4.83 ± 0.13c	6.27 ± 0.23b
	NO_3_^−^-N (mg/kg)	0.26 ± 0.02b	0.45 ± 0.04a	0.22 ± 0.02b	0.31 ± 0.05b
	SOC (g/kg)	21.25 ± 1.58a	24.38 ± 2.06a	19.75 ± 1.51b	20.19 ± 1.98b
*Tilia amurensis*	pH	5.04 ± 0.52a	4.88 ± 0.27b	5.17 ± 0.28a	4.79 ± 0.29b
	SWC(%)	52.26 ± 5.37a	54.33 ± 4.35a	47.28 ± 3.26b	45.28 ± 3.49b
	NH_4_^+^-N (mg/kg)	5.18 ± 0.32b	7.37 ± 0.12a	4.17 ± 0.16b	5.09 ± 0.23b
	NO_3_^−^-N (mg/kg)	0.31 ± 0.03b	0.52 ± 0.05a	0.28 ± 0.01c	0.39 ± 0.04b
	SOC(g/kg)	22.52 ± 2.18b	25.97 ± 2.04a	21.71 ± 1.99b	22.05 ± 2.83b
*Fraxinus mandshurica*	pH	5.46 ± 0.42b	5.02 ± 0.29c	5.96 ± 0.31a	5.45 ± 0.43b
	SWC(%)	51.38 ± 3.07a	54.17 ± 5.08a	46.38 ± 5.25b	47.02 ± 4.38b
	NH_4_^+^-N (mg/kg)	5.29 ± 0.48b	7.03 ± 0.35a	4.45 ± 0.16c	5.57 ± 0.27b
	NO_3_^−^-N (mg/kg)	0.29 ± 0.02c	0.43 ± 0.04a	0.26 ± 0.01c	0.35 ± 0.04b
	SOC(g/kg)	21.49 ± 2.21a	23.83 ± 2.67a	19.58 ± 1.63b	20.99 ± 1.87b

Note: CK represents the control, + N represents the N addition with 50 kg N·ha ⁻ ¹·yr ⁻ ¹, -P represents precipitation decreases by 30%, + N-P represents nitrogen-water interaction, SWC represents soil water content, NH_4_^+^-N represents soil NH_4_^+^, NO_3_^−^-N represents soil NO_3_^−^, SOC represents soil organic carbon. Significant differences between treatments are indicated by different lowercase letters (p < 0.05).

### 4.2. GRSP and soil aggregate dynamics

**Fig 1:**
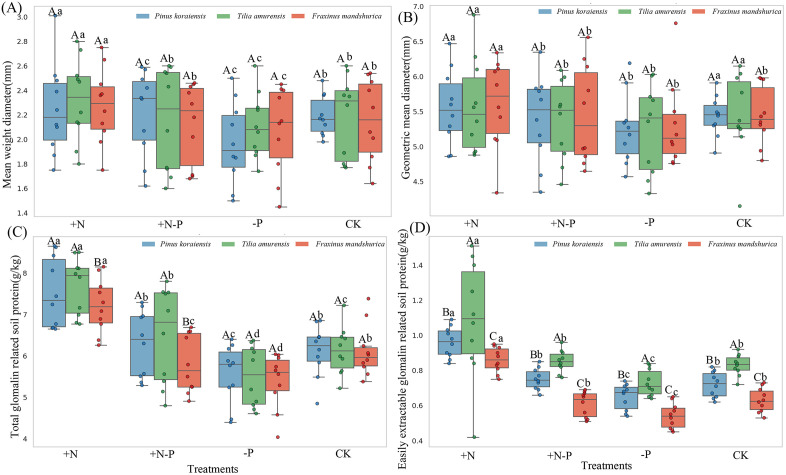
Changes of mean weight diameter (A), geometric mean diameter (B), total glomalin related soil protein (C), easily extractable glomalin related soil protein (D) in diferents treatments. Note: CK represents the control treatment, + N represents the N addition with 50 kg N·ha ⁻ ¹·yr ⁻ ¹, -P represents precipitation decreases by 30%, + N-P represents nitrogen-water interaction. Different lowercase letters indicate signifcant diferences between diferent treatments, while different capital letters denote statistically significant differences within the same treatment across different species (*Pinus koraiensis*, *Tilia amurensis* and *Fraxinus mandshurica*). A significant difference is indicated when P < 0.05.

Soil aggregate properties and GRSP exhibited significant variations across treatments and species (Fig 1). MWD was significantly higher in the + N and CK treatments compared to the -P treatment across all species (Fig 1A). GMD was significantly higher in the + N treatment than in the -P treatment in all species (Fig 1B). T-GRSP content was significantly higher in the + N treatment and significantly lower in the -P treatment across all species (Fig 1C). EE-GRSP was significantly higher in the + N treatment compared to other treatments across all species, whereas it was significantly lower in the -P and +N-P treatments (Fig 1D).

### 4.3. The influence of SOC on the characteristics of soil aggregates and GRSP

**Fig 2:**
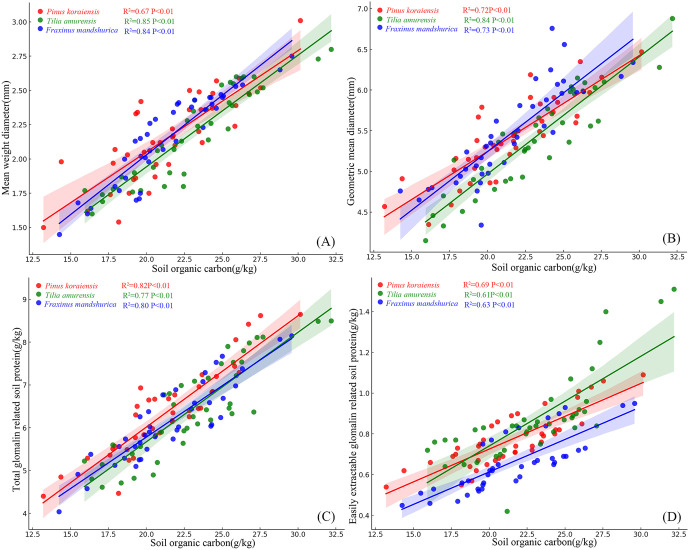
Relationships between soil organic carbon and mean weight diameter (A), geometric mean diameter (B), total glomalin related soil protein (C), easily extractable glomalin related soil protein (D).

A significant positive correlation was observed between SOC and soil aggregate characteristics, as well as GRSP across all species (Fig 2). MWD exhibited a significant positive correlation with SOC across all species (Fig 2A). Similarly, GMD showed a significant positive correlation with SOC across all species (Fig 2B). T-GRSP was significantly positively correlated with SOC across all species (Fig 2C). Likewise, EE-GRSP exhibited a significant positive correlation with SOC across all species (Fig 2D).

### 4.4. Variation in soil aggregate composition

**Fig 3:**
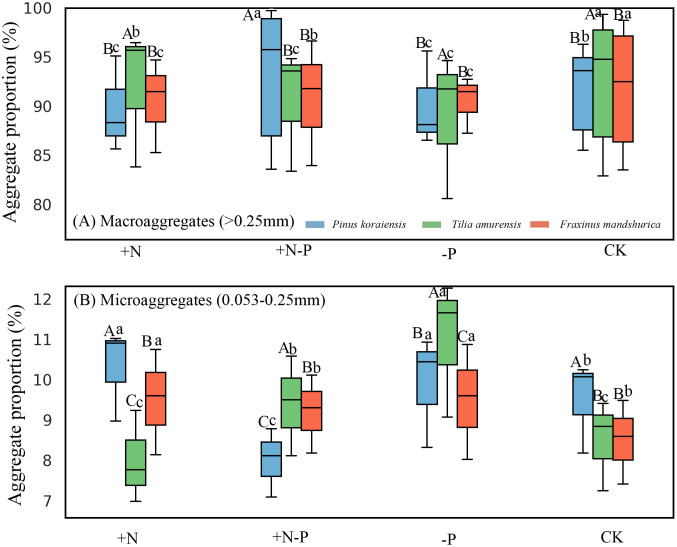
Soil aggregate distribution under different nitrogen and precipitation treatments in three shrub species. Note: (A) Macroaggregates (> 0.25 mm) and (B) Microaggregates (0.053–0.25 mm) represent the proportional contribution of each aggregate fraction to total soil aggregates. CK represents the control treatment, + N represents nitrogen addition with 50 kg N ha ⁻ ¹ yr ⁻ ¹, –P indicates a 30% reduction in precipitation, and +N–P represents the combined nitrogen–water interaction. Different uppercase letters indicate significant differences among treatments within the same species, and different lowercase letters indicate significant differences among species within the same treatment at p < 0.05 (based on Tukey’s HSD test).

Aggregate proportions of both macroaggregates (>0.25 mm) and microaggregates (0.053–0.25 mm) varied significantly among treatments and species. Overall, CK maintained significantly higher proportions than +N and +N–P across all species, whereas –P consistently resulted in significantly lower values (Fig 3). Across species, Pinus koraiensis generally exhibited significantly higher aggregate proportions than Tilia amurensis and Fraxinus mandshurica under CK (Fig 3A), while T. amurensis showed significantly higher values under –P and significantly lower values under +N–P (Fig 3B). F. mandshurica maintained intermediate proportions but exhibited significantly lower values than the other species under +N–P (Fig 3).

SOC was significantly negatively correlated to the proportion of 0.053–0.25 mm aggregates. MWD and GMD exhibited a significant positive correlation with macroaggregates (4–8 mm, 2–4 mm, 1–2 mm) but were significantly negatively correlated to microaggregates (0.25–1 mm, 0.053–0.25 mm) (Table 3). T-GRSP showed a significant positive correlation with macroaggregates (4–8 mm, 2–4 mm, 1–2 mm) and a significant negative correlation with microaggregates (0.25–1 mm, 0.053–0.25 mm). EE-GRSP was significantly positively correlated to 4–8 mm aggregates but negatively correlated to 0.25–1 mm and 0.053–0.25 mm aggregates (Table 3). The treatment had a significant impact on soil aggregate distribution, while species had no significant effect. The interaction between species and treatment significantly influenced the distribution of 4–8 mm, 1–2 mm, and 0.25–1 mm aggregates but had no significant effect on other aggregate sizes (Table 3).

**Table 3:** Pearson’s correlation analysis between soil aggregate size andMWD, GMD, SOC, T – GRSP, EE – GRSP.

Type	4-8 mm	2-4 mm	1-2 mm	0.25-1 mm	0.053-0.25 mm
SOC	0.327	0.243	0.228	−0.323	−0.694**
MWD	0.583**	0.490*	0.489*	−0.503*	−0.567**
GMD	0.643**	0.674**	0.585**	−0.435*	−0.606**
T-GRSP	0.618**	0.611**	0.512**	−0.566**	−0.712**
EE-GRSP	0.348*	0.278	0.232	−0.341*	−0.566**
Treatments	*	*	*	*	NS
Species	NS	NS	NS	NS	NS
Species×Treatments	*	NS	*	*	NS

Note: * p < 0.05; ** p < 0.01. Shown are P value of the respective variables and the model itself. CK represents the control, + N represents the N addition with 50 kg N·ha ⁻ ¹·yr ⁻ ¹, -P represents precipitation decreases by 30%, + N-P represents nitrogen-water interaction, SOC represents soil organic carbon, MWD represents mean weight diameter, GMD represents geometric mean diameter, T-GRSP represents total glomalin related soil protein, EE-GRSP represents Easily extractable glomalin related soil protein.

### 4.5. The relationship between soil aggregate, GRSP and environmental factors

**Fig 4:**
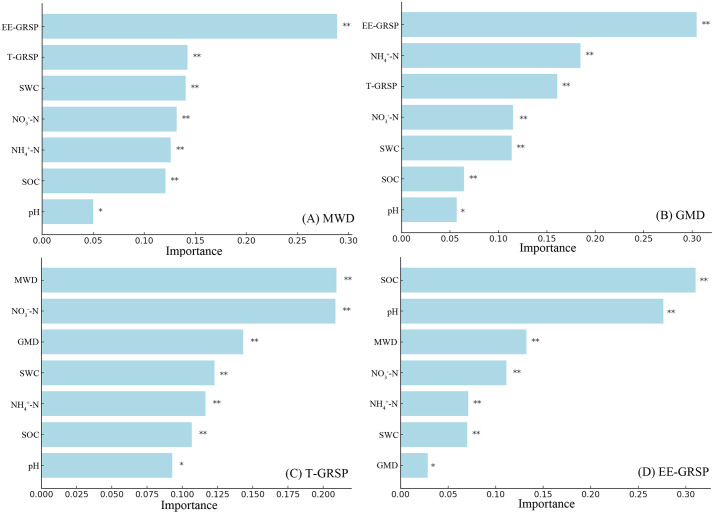
Random forest variable importance plot. The variables are ranked in order of relevance in predicting soil aggregate stability (A MWD, B GMD, C T-GRSP, D EE-GRSP). Note: The importance measure considered for the analysis is the mean decrease in accuracy computed via random forest classifcation algorithm. SOC represents soil organic carbon, SWC represents soil water content, MWD represents mean weight diameter, GMD represents geometric mean diameter, NH_4_^+^ -N represents soil NH_4_^+^, NO_3_^−^-N represents soil NO_3_^−^, T-GRSP represents total glomalin related soil protein, EE-GRSP represents easily extractable glomalin related soil protein. A single asterisk (*) represents p < 0.05, double asterisks (**) represent P < 0.01.

Random forest modeling identified significant differences in the influence of environmental factors on soil aggregate stability and GRSP (Fig 4). EE-GRSP had the highest importance in predicting MWD and GMD, followed by NH₄ ⁺ -N and T-GRSP (Fig 4A, B). For T-GRSP, MWD had the highest importance, followed by NO₃ ⁻ –N and GMD (Fig 4C). For EE-GRSP, SOC had the highest importance, followed by pH and MWD (Fig 4D).

**Fig 5:**
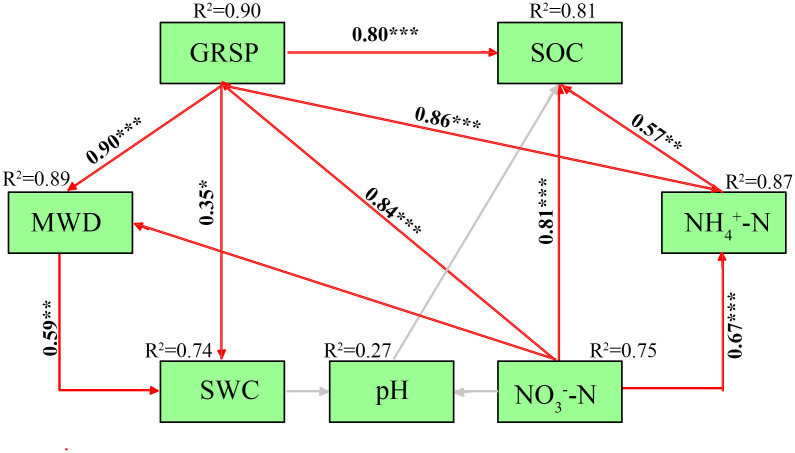
Structural equation model of soil environmental factors, GRSP and MWD. Note: The red arrow indicates positive correlation, the grey arrow indicates no signifcant correlation. SOC represents soil organic carbon, MWD represents mean weight diameter, SWC represents soil water content, GRSP represents glomalin related soil protein, NH_4_^+^ -N represents soil NH_4_^+^, NO_3_^−^-N represents soil NO_3_^−^. **p < 0.01, ***p < 0.001. Model fit indices: χ²/df = 1.82, CFI = 0.963, RMSEA = 0.045, SRMR = 0.031, indicating an excellent model fit (p < 0.001).

The structural equation model revealed significant relationships among soil environmental factors, GRSP, and MWD (Fig 5). GRSP was significantly positively correlated to MWD and SOC (Fig 5). SOC was significantly positively correlated to NH₄ ⁺ -N and NO₃ ⁻ -N. NH₄ ⁺ -N was significantly positively correlated to NO₃ ⁻ -N (Fig 5). SWC exhibited a significant positive correlation with MWD and GRSP. Additionally, NO₃ ⁻ -N was significantly positively correlated to SOC (Fig 5).

## 5. Discussion

### 5.1. Soil aggregation responses mediated by species-specific GRSP regulation under altered N and water regimes

The observed shifts in GRSP and aggregate-size distribution under nitrogen addition and precipitation reduction provide compelling evidence that tree species differentially regulate soil physical integrity through GRSP-associated pathways (Figs 1C, D; 3; Table 3). Across all species, nitrogen addition significantly enhanced both T-GRSP and EE-GRSP, coinciding with higher proportions of macroaggregates (>0.25 mm) and increased aggregate stability, as indicated by MWD and GMD (Figs 1A, B; 3). In contrast, precipitation reduction suppressed GRSP levels and decreased aggregate proportions, particularly in *F. mandshurica* and *T. amurensis* (Fig 3B). These patterns support a species-dependent mechanism whereby differential GRSP production modulates soil aggregate architecture under altered nutrient and moisture conditions [28,29].

The interspecific variation in GRSP responses was especially pronounced. In *P. koraiensis*, nitrogen enrichment markedly stimulated GRSP accumulation and maintained higher proportions of macroaggregates, whereas *F. mandshurica* and *T. amurensis* exhibited reductions in GRSP production and aggregate stability under combined nitrogen–water stress (+N–P) (Fig 3). This divergence likely reflects differences in root functional traits and belowground carbon allocation under environmental constraints [30,31]. Species-specific differences in root morphology, exudate chemistry, and mycorrhizal association patterns may influence GRSP secretion and consequently reshape aggregate-size distribution [32,33].

The structural relationships observed further highlight a mechanistic pathway whereby root-associated regulation of GRSP production governs aggregate stability. Correlation analysis (Table 3) revealed that both T-GRSP and EE-GRSP were positively correlated with macroaggregates (>0.25 mm) and negatively correlated with microaggregates (0.053–0.25 mm), supporting the hypothesis that GRSP serves as a key binding agent in macroaggregate formation [34]. Moreover, significant interaction effects between species identity and treatment type were observed for specific aggregate fractions (Fig 3), indicating that environmental modulation of GRSP is species-dependent and not uniform across taxa [35].

These findings validate our initial hypothesis and underscore that tree species regulate GRSP-associated soil aggregation in divergent ways under nitrogen enrichment and precipitation reduction [36,37]. The enhanced macroaggregation in *P. koraiensis* under nitrogen addition and the weakened aggregation in *F. mandshurica* and *T. amurensis* under precipitation reduction reflect trait-based differences in environmental responsiveness. This functional differentiation emphasizes the role of species identity in driving soil structural dynamics under concurrent hydrological and nutrient perturbations [38,39]. Integrative models incorporating GRSP dynamics, root traits, and aggregate stability could improve predictions of soil resilience under global change. From a management perspective, promoting functional diversity in tree species composition may enhance forest ecosystem stability in response to shifting precipitation and nitrogen regimes. Despite the strong relationships observed between GRSP fractions and soil aggregate stability, we acknowledge several methodological limitations associated with the interpretation of GRSP as an indicator of arbuscular mycorrhizal activity. First, GRSP represents an operationally defined protein pool and does not directly quantify AMF colonization, hyphal biomass, or molecular abundance. Therefore, the species-specific differences reported here should be understood as indirect reflections of belowground fungal processes rather than direct measurements of AMF structures. Second, Bradford-based GRSP assays can extract non-glomalin proteins and humic-derived substances, introducing uncertainty into the biochemical specificity of GRSP [25]. This methodological constraint does not undermine the observed ecological patterns but highlights that GRSP should be interpreted as a functional soil protein fraction associated with AMF-related processes rather than a taxonomically precise AMF biomarker. In addition, part of the species-specific variation observed here may reflect unmeasured fine-root turnover or microsite heterogeneity, which could interact with GRSP production and aggregation responses and should be examined in future studies.

### 5.2. SOC and moisture synergistically enhance EE-GRSP pathways to stabilize soil structure

Soil organic carbon (SOC) and soil water content (SWC) emerged as the dominant abiotic drivers of EE-GRSP accumulation and macroaggregate stability, supporting our second hypothesis [40,41]. Positive associations between SOC and both MWD and GMD (Fig 2A, B), along with strong correlations between SOC and GRSP fractions (Fig 2C, D), indicate that carbon availability acts as a central driver of both microbial secretion and soil aggregation. Structural equation modeling further demonstrated that SOC and SWC synergistically stimulated EE-GRSP production, which subsequently enhanced MWD (Fig 5), outlining a clear carbon–water–microbe–structure coupling pathway. SOC and SWC regulate fungal physiology and EE-GRSP secretion, which in turn mediates the biological reinforcement of soil aggregates [42,43]. These findings were corroborated by random forest models, which ranked EE-GRSP and SOC as the top predictors of MWD and GMD (Fig 4A, B), and confirmed EE-GRSP as the most responsive fraction to SOC and soil physicochemical traits (Fig 4D). These coordinated effects are consistent with prior findings showing that both resource availability and environmental moisture regulate arbuscular mycorrhizal metabolism and glomalin output.

This microbial-mediated mechanism is biologically plausible given that EE-GRSP, a thermostable glycoprotein exuded by AMF, is tightly regulated by fungal metabolism and carbon fluxes [42]. Elevated SOC likely enhances AMF biomass and exudation, increasing the deposition of EE-GRSP at the root–soil interface and promoting particle binding [43]. The functional evidence from Table 3, showing positive associations between EE-GRSP and macroaggregate proportions and negative associations with microaggregates, further underscores the hierarchical stabilization function of this glycoprotein. Moreover, the significant influence of SWC and inorganic N (NH₄ ⁺ –N, NO₃ ⁻ –N) on GRSP accumulation (Fig 5) aligns with previous findings that both moisture and N availability regulate fungal colonization and polysaccharide production [44,45].

The dominant role of EE-GRSP over NH₄ ⁺ -N and T-GRSP in predicting MWD (Fig 4A) suggests that rapidly cycling, bioavailable protein fractions exert greater structural control than more recalcitrant pools [46]. This insight is consistent with prior studies highlighting the pivotal role of active glomalin in short-term aggregation [47,48], and supports a functional distinction between EE-GRSP and total GRSP in driving structural transitions. These results thus integrate multiple environmental drivers—SOC, water, and N—into a unified mechanism governing microbial regulation of soil structure, confirming that biogeochemical context modulates fungal exudation pathways and structural outcomes [49]. Nevertheless, the SEM-based pathway represents an inferred mechanism rather than a direct measurement of microbial processes, and the relative contributions of AMF physiology versus broader microbial communities cannot be fully resolved without molecular or isotopic evidence

By highlighting a microbially centered mechanism linking SOC availability to soil physical resilience, this study offers important ecological and management implications [50]. The demonstrated SOC–SWC–N–GRSP pathway represents a sensitive indicator system under scenarios of global change, where nutrient deposition and hydrological stress may interact to destabilize soil aggregates. Management strategies aimed at increasing SOC stocks—such as organic mulching, litter retention, or native vegetation restoration—could enhance EE-GRSP secretion and thus reinforce structural integrity in vulnerable landscapes. Future work should incorporate isotopic tracing of GRSP-C fluxes and microbial functional profiling to disentangle the metabolic and taxonomic pathways that underlie carbon-mediated structural stabilization.

### 5.3. Trait–environment coupling controls soil aggregation pathways under climate and nutrient perturbations

Together, the findings across species- and trait-based responses converge on a unifying mechanism whereby species-specific physiological traits and resource availability—particularly carbon and water—jointly mediate glomalin production and soil aggregation [51,52]. The divergent aggregation patterns observed among the three tree species under N addition and precipitation reduction, along with the strong influence of SOC and EE-GRSP on macroaggregate formation, highlight a dual regulatory axis: trait-driven modulation of belowground carbon allocation and environment-induced shifts in microbial exudation. This integrated mechanism underscores the necessity of considering both biotic functional differentiation and abiotic resource dynamics in predicting soil structural change [53,54].

These insights hold important implications for forest ecosystem resilience under global environmental change. Conventional soil–vegetation models rarely incorporate fungal-derived proteins or species-specific belowground traits as dynamic drivers of soil structural stability [55]. Our results suggest that SOC, soil moisture, and trait-mediated GRSP production should be explicitly represented in such models, not merely as background variables but as interactive factors influencing the biological reinforcement of soil aggregates. Furthermore, the identification of EE-GRSP as a rapidly responsive, microbially regulated protein fraction with strong predictive capacity for aggregate stability offers a sensitive bio-indicator for monitoring structural vulnerability under nutrient and hydrological perturbations [56,57].

To enhance predictive capacity and ecological relevance, future studies should extend beyond short-term evaluations and incorporate seasonal to decadal timescales to capture the temporal dynamics of GRSP accumulation and aggregate turnover [58,59]. Mechanistically, coupling GRSP measurements with extracellular enzyme activity and microbial functional diversity indices may help disentangle the biochemical pathways underlying protein secretion and aggregate stabilization [60]. Additionally, integrating trait-based models of species-specific carbon inputs with parameterized functions of EE-GRSP production would enable the development of next-generation models that link plant identity, microbial metabolism, and soil structure under changing environmental regimes [61]. Because the study was conducted within a single mixed-forest ecosystem, the generality of these trait–environment pathways may vary across soil types, stand ages, and climatic conditions, which limits direct extrapolation to other forest systems. Furthermore, precipitation reduction was applied at the main-plot level (n = 3), which constrains statistical power for precipitation effects and represents an inherent limitation of long-term forest manipulation experiments. This functional synthesis provides a robust framework for understanding and managing the physical integrity of forest soils in response to accelerated global change [62].

## 6. Conclusion

This study demonstrates that soil aggregate stability in temperate forest ecosystems is jointly regulated by species identity and resource availability, with significant implications for microbial processes and carbon-mediated soil resilience. We identified four key conclusions: (i) Tree species differed in their ability to stimulate glomalin secretion and promote macroaggregate formation, with *P. koraiensis* showing greater enhancement of EE-GRSP and structural stability under N enrichment, while *T. amurensis* and *F. mandshurica* contributed less under drought conditions. (ii) SOC and SWC emerged as dominant abiotic regulators of EE-GRSP production, forming a carbon–water–microbe–structure coupling pathway. Structural equation modeling confirmed that SOC and SWC synergistically stimulated EE-GRSP secretion, which in turn significantly enhanced macroaggregate stability. (iii) EE-GRSP, as a rapidly cycling and bioavailable protein fraction, exerted a stronger influence on soil aggregation than T-GRSP or inorganic N, underscoring the pivotal role of active microbial products in determining soil physical structure. (iv) Trait-mediated species effects and resource-driven microbial exudation together defined a dual regulatory axis of soil aggregation, emphasizing that both plant functional identity and environmental resource fluxes must be jointly considered in models of soil structural dynamics under global change scenarios.

These findings contribute to a mechanistic understanding of how species-specific regulation and microbial activity underpin soil aggregation processes. Forest management should prioritize SOC accumulation, moisture conservation, and species selection based on functional traits that promote microbial exudation and macroaggregate formation to enhance long-term soil resilience. The identification of EE-GRSP as a sensitive and responsive bioindicator provides a practical tool for assessing structural vulnerability under interacting nutrient and hydrological stresses.

Future research should quantify the temporal dynamics of GRSP production and turnover through long-term field observations, while integrating microbial enzyme activity, isotopic tracing, and trait-based analysis into predictive models. Such process-explicit and trait-informed frameworks are essential to forecast how biotic–abiotic interactions mediate soil structural transitions and ecosystem function under accelerating global environmental change.

## Supporting information

S1. Data Raw data for the figures and tables in the manuscript. (XLSX)
